# Modulation of Ion Transport Across Plant Membranes by Polyamines: Understanding Specific Modes of Action Under Stress

**DOI:** 10.3389/fpls.2020.616077

**Published:** 2021-01-26

**Authors:** Igor Pottosin, Miguel Olivas-Aguirre, Oxana Dobrovinskaya, Isaac Zepeda-Jazo, Sergey Shabala

**Affiliations:** ^1^International Research Centre for Environmental Membrane Biology, Foshan University, Foshan, China; ^2^Biomedical Center, University of Colima, Colima, Mexico; ^3^Food Genomics Department, Universidad de La Ciénega del Estado de Michoacán de Ocampo, Sahuayo, Mexico; ^4^Tasmanian Institute for Agriculture, College of Science and Engineering, University of Tasmania, Hobart, TAS, Australia

**Keywords:** abiotic stress, Ca^2+^ ATPase, H^+^-ATPase, ion channel, organelle, polyamines, TPC1, vacuole

## Abstract

This work critically discusses the direct and indirect effects of natural polyamines and their catabolites such as reactive oxygen species and γ-aminobutyric acid on the activity of key plant ion-transporting proteins such as plasma membrane H^+^ and Ca^2+^ ATPases and K^+^-selective and cation channels in the plasma membrane and tonoplast, in the context of their involvement in stress responses. Docking analysis predicts a distinct binding for putrescine and longer polyamines within the pore of the vacuolar TPC1/SV channel, one of the key determinants of the cell ionic homeostasis and signaling under stress conditions, and an additional site for spermine, which overlaps with the cytosolic regulatory Ca^2+^-binding site. Several unresolved problems are summarized, including the correct estimates of the subcellular levels of polyamines and their catabolites, their unexplored effects on nucleotide-gated and glutamate receptor channels of cell membranes and Ca^2+^-permeable and K^+^-selective channels in the membranes of plant mitochondria and chloroplasts, and pleiotropic mechanisms of polyamines’ action on H^+^ and Ca^2+^ pumps.

## Introduction

Polyamines (PAs) are plant growth regulators and important components of plant stress responses (Alcázar et al., 2010; Takahashi and Kakehi, 2010; Pottosin and Shabala, 2014; Pál et al., 2015; Paschalidis et al., 2019). PAs putrescine^2+^ (Put), spermidine^3+^ (Spd), and spermine^4+^ (Spm) are natural polycations and, therefore, can affect different cation transporters, including those regulating Ca^2+^ homeostasis and signaling (Pottosin and Shabala, 2014). Stress-induced PA changes can remodel ion transport across cellular membranes, with important consequences for plant performance (Pottosin and Shabala, 2014; Pottosin, 2015). PA effects on ion transport depend on cell-, tissue-, and genotype-specificity and growth conditions (Pandolfi et al., 2010). Most likely, PAs were not originally designed as signaling compounds but then acquired this function during evolution by leveraging on a strong variation of levels of individual PAs and their catabolites during plant responses to changing environment. Owing to this level of complexity, the initial question should be related to the primary effects of different PAs and their catabolites (e.g., ROS) on individual ion transporters. Are these effects direct and specific for different PAs? Are there allosteric PA-binding sites? *In vivo* effects may be also dependent on the PA metabolism and traffic, and an involvement of the intermediate signaling. Finally, catabolization of PAs and signaling by their catabolites, ROS and GABA, should be considered. The aim of this work is to elucidate diverse effects of PAs on key plant membrane ion transporters to stimulate more focused studies in this field.

## Polyamine Transport May Affect Membrane Potential and ΔpH

Polyamines induce a rapid depolarization in roots and leaves (Fromm et al., 1997; Ozawa et al., 2010; Pottosin et al., 2014a). A significant part of it is due to the traffic *per se* of these polycations across the plasma membrane, PM (Pottosin et al., 2014a). Interestingly, PM Put transporter, PUT3, is phosphorylated by SOS2 (CIPK24). It forms a tertiary complex with SOS1 (PM Na^+^/H^+^ antiporter) and SOS2, key elements in response to salinity; within this complex, the activity of PUT3 and SOS1 is synergistically modulated (Chai et al., 2020). Thus, Put uptake can contribute to the pH and Na^+^ regulation. PA traffic is documented for most intracellular membranes, albeit transporters, which facilitate PA uptake into plant vacuoles and mitochondria, remain elusive (Fujita and Shinozaki, 2015). No specific PA transporter was postulated for chloroplasts, but chloroplasts represent the main source of Put synthesis in photosynthetic tissues (Borrell et al., 1995). In *Arabidopsis* chloroplasts, Put may account for up to 40% of the total cellular Put (Krueger et al., 2011). A small but significant fraction of unprotonated (uncharged) Put can freely diffuse through the thylakoid membrane and partly buffer the light-induced thylakoid lumen acidification, changing the proportion between membrane potential (ΔΨ) and ΔpH across the thylakoid membrane (Ioannidis et al., 2006, 2012). Under salinity, Put-induced increase in ΔΨ at the expense of ΔpH stimulates cyclic electron flow and decreases pH-dependent non-photochemical quenching. This increases the quantum yield by PSII, decreasing the overreduction of PSI acceptors and, eventually, enhancing the ATP production (Wu X. et al., 2019). A stark increase in the Put production upon K^+^ deficiency is accompanied by an increase in the Mg^2+^ content. Mg^2+^ uptake into thylakoids causes an opposite effect on ΔΨ/ΔpH partition and favors lumen acidification and stromal alkalinization, optimal for the Calvin cycle (Cui et al., 2020).

## Direct Effects of Polyamines on the Tonoplast Cation Channels

Tonoplast harbors two types of cation channels: slow (SV/TPC1) and fast (FV) vacuolar channels. FV channels are inhibited by micromolar Ca^2+^ and Mg^2+^ and conduct small monovalent cations indiscriminately, whereas SV channels are activated by an elevated cytosolic Ca^2+^ and conduct both monovalent and divalent cations with a little preference (Pottosin and Dobrovinskaya, 2014, 2018). Both channels are rapidly, reversibly, and directly suppressed by PAs (Pottosin and Shabala, 2014; Table 1). PAs cause the voltage-dependent block of the SV/TPC1 pore. In agreement with an early study (Colombo et al., 1992), PAs can be transported by SV/TPC1. The FV current is intrinsically flickering, and its inhibition by cytosolic PAs is voltage independent. It remains to be elucidated whether PAs block the FV channel or alter its gating.

**TABLE 1:** Key plant plasma membrane and vacuolar ion transporters—targets for polyamines.

**Ion transporter**	**Ionic transport function/physiological implications**	**Involvement in stress responses**	**PA effect (half-efficient concentration)**
Slow vacuolar SV channel (TPC1)	Transport of Ca^2+^, Na^+^, Mg^2+^ K^+^, NH_4_^+^, and other small cations across tonoplast ^(1)^, vacuolar K^+^ release/cytoplasmic K^+^ homeostasis ^(2)^, Ca^2+^- and voltage-induced vacuole electrical excitability/intracellular signaling ^(3,4)^, Ca^2+^ release and long-distance signaling/sensors and amplifiers of Ca^2+^ signal, ROS, and Ca^2+^ interplay ^(3,5–7)^, cation homeostasis ^(8,9)^, ABA signaling in seed germination and stomatal closure ^(10,11)^	Salt stress (detrimental vacuolar Na^+^ leak) ^(3,5,7,12–14)^, long-distance signaling ^(5)^, aluminum tolerance ^(15)^, lead stress response^ (16)^, wounding via jasmonate signaling^ (17)^, insect herbivory response ^(6)^, hypoxia sensors ^(18)^	Direct, reversible. Voltage-dependent block from either membrane side (Spm^4+^ 50 μM > Spd^3+^ 500 μM > Put^2+^ 3 mM)^ (19–21)^
Fast vacuolar FV channel (unknown molecular identity)	NH_4_^+^ > K^+^ > Na^+^ transport ^(22)^, K^+^ uptake and release/Volume adjustment via auxin ^(23)^, tonoplast electric potential regulation^ (24)^, K^+^ sensing/intracellular K^+^ homeostasis ^(25)^, vacuolar volume adjustment via auxin ^(26)^	Detrimental for salt stress (vacuolar Na^+^ leak) ^(13,24,27)^, K^+^ deprivation (vacuolar K^+^-cycling) ^(25,28)^	Direct, reversible, voltage-independent, from the cytosolic side (Spm^4+^ 6 μM > Spd^3+^ 80 μM >> Put^2+^ 4 mM) ^(19,21,29)^
Vacuolar K^+^ VK channel (TPK1)	Selective K^+^ transport across tonoplast ^(30–33)^, K^+^ homeostasis, vacuolar K^+^ mobilization, stomata closure^ (34–36)^, mechano- and osmo-sensor^ (37)^, vacuolar excitability in combination with TPC1^(4)^	Salt stress ^(31,33,38)^ and K^+^ deprivation ^(28,34,36)^, tolerance by refilling of cytosolic K^+^	Voltage-independent, cytosolic side block (Spm^4+^ ∼ Spd^3+^ 0.5 mM)^ (39)^
Inward-rectifying shaker K^+^ channels (AKT1, KAT1)	K^+^ uptake and tissue K^+^ transport^ (33,40–44)^ and stomata opening ^(45,46)^	K^+^ starvation/deficiency, K^+^ uptake^ (33,47,48)^, Fe^2+^ toxicity tolerance ^(49)^, drought tolerance by enhancing root K^+^ uptake ^(42,50,51)^	Indirect inhibition, cytosolic side ^(52)^, indirect, extracellular side^ (53,54)^, voltage-independent (Spm^4+^ ∼ Spd^3+^ ∼ Put^2+^ 0.5 –1 mM), inhibition via increase in PIP_2_^(55)^
Outward-rectifying shaker K^+^ Channels (SKOR, GORK)	K^+^ efflux, general metabolic switch, cell death^ (33,56,57)^, K^+^ loading into xylem, SKOR ^(43,44,58)^, stomata closure, GORK ^(59,60)^, initial depolarization phase of the action potentials ^(61)^	Salt stress (sensitivity via K^+^ loss) ^(43,57,58,62,63)^, cell death^ (64)^, relocation of energy from metabolism to defense^ (56)^, oxidative stress tolerance by cation distribution ^(65)^	Indirect inhibition from extracellular side, voltage-independent (Spm^4+^ ∼ Put^2+^ 1 mM)^ (54)^, activation from extracellular side via increase in PIP_2_ and PLDδ activity (Spm^4+^ = tSpm^4+^ > Spd^3+^ > > Put^2+^≈ Dap^2+^) ^(66,67)^, GABA formed by Put^2+^ catabolism decreases the expression ^(63)^
Voltage-independent non-selective cation channels VI-NSCC (uncertain molecular identity)	General cation transport/uptake of nutrients, growth and development, Ca^2+^ influx/transduction of stimuli (cyclic nucleotides, membrane stretch, amino acids, and purines) ^(1,68)^	Salt stress sensitivity, Na^+^ influx^ (1,68–71)^	Extracellular side, voltage-independent inhibition (Spm^4+^ ∼ Spd^3+^ (∼1 mM) > Put^2+^)^ (53)^, extracellular side, indirect? (Spm^4+^ ∼ Spd^3+^ ∼ Put^2+^ 1 mM) ^(72^.^73)^
Reactive oxygen species-induced Ca^2+^ influx ROSIC Weakly voltage-dependent, OH•-induced non-selective conductance (unknown molecular identity)	Non-selective cation and small anions conductance^ (74–77)^, Ca^2+^ influx and signaling via ROS and ABA/stomatal closure^ (75,76,78)^, polarized (root hair, pollen tube) growth^ (74,79)^	Drought stress, signaling^ (78)^, salinity ^(64)^, hypersensitive response to biotic stress ^(80)^	Extracellular PAs act as cofactors for ROSIC activation by OH (Spm^4+^ ∼ Spd^3+^ ∼ Put^2+^ 1 mM)^ (75,81,82)^, PA export and oxidation by DAO or PAO results in the ROS burst in the apoplast and activation of Ca^2+^ influx via ROSIC upon pathogen attack ^(83–85)^, salinity ^(86)^, ABA-induced stomata closure ^(87)^
H^+^-pump P-type ATPase (AHA: auto inhibited H^+^-ATPAse)	Drives H^+^ extrusion and PM hyperpolarization^ (88,89)^, intracellular and apoplastic pH regulation^ (90–94)^, fueling H^+^-coupled secondary transports ^(95–97)^, plant growth and development, stomatal aperture^ (98–101)^	Salt stress, generation of electric and pH gradients for K^+^ uptake ^(43,62,102–105)^, Al^3+^ toxicity/tolerance ^(106,107)^, pathogen infection sensitivity ^(97)^, alkaline stress tolerance ^(108,109)^, drought adaptation by auxin ^(110)^, wounding, leaf-to-leaf electrical signaling and plant defense ^(111)^	*Activation/Stimulation* Rapid activation (Spm^4+^ 0.1 mM, Spd^3+^ or Put^2+^ 1mM) ^(112)^, (Put^2+^ in the elongation zone) ^(113)^, (Spm^4+^ ∼ Spd^3+^ ∼ Put^2+^ ∼1mM)^ (114)^, (Spm^4+^ > Spd^3+^ > Put^2+^ 1 mM), ^(80)^ via 14-3-3 proteins binding (Spm^4+^ only, ∼0.1mM)^ (115)^, via GABA by Put^2+^ catabolism^ (63)^ *Inhibition/Suppression* Rapid (Spm^4+^ 1 mM) ^(112,113)^, long-term suppression/decrease in expression (Put^2+^∼ Spm^4+^ ∼ Spd^3+^ 50 μM) ^(116)^ *Restoration* Long term (Put^2+^ or Spd^3+^ 0.5–1 mM and Spm^4+^ 1 mM) ^(102,117)^
Ca^2+^-pump P-type ATPase PMCA (the plasma membrane located Ca^2+^-ATPases) Type IIB (ACA: auto-inhibited Ca^2+^-ATPase)	Ca^2+^ extrusion from cytosol, Ca^2+^ signaling, protein glycosylation, protein and polysaccharide secretion, enzymes activation, stomatal closure ^(118–123)^, plant growth and development ^(124–126)^	Abiotic stresses^ (118,127)^, Ca^2+^ stress (deficiency) ^(128)^, salinity adaptation ^(118,119)^, chilling ^(129)^, anoxia ^(118)^, Mn^2+^ toxicity ^(119)^, pathogen-induced cell death and signaling ^(130)^	Rapid activation (Spm^4+^ ∼ Put^2+^ 0.1–1 mM) ^(75,81,82,119)^ and long-term potentiation via Spm^4+^, Spd^3+^, or Put^2+^ production by salicylic acid^ (131)^

*^1^Pottosin and Dobrovinskaya, 2014; ^2^Hedrich et al., 2018; ^3^Pottosin and Dobrovinskaya, 2018; ^4^Jaślan et al., 2019; ^5^Choi et al., 2014; ^6^Kiep et al., 2015; ^7^Evans et al., 2016; ^8^Hedrich and Marten, 2011; ^9^Hedrich et al., 2018; ^10^Peiter et al., 2005; ^11^Peiter, 2011; ^12^Bonales-Alatorre et al., 2013; ^13^Shabala et al., 2020; ^14^Koselski et al., 2019; ^15^Wherrett et al., 2005; ^16^Miśkiewicz et al., 2020; ^17^Lenglet et al., 2017; ^18^Wang et al., 2017; ^19^Dobrovinskaya et al., 1999b; ^20^Dobrovinskaya et al., 1999a; ^21^Pottosin and Shabala, 2014; ^22^Brüggemann et al., 1999; ^23^Burdach et al., 2020; ^24^Tikhonova et al., 1997; ^25^Pottosin and Martínez-Estévez, 2003; ^26^Burdach et al., 2018; ^27^Pottosin and Muñiz, 2002; ^28^Cui et al., 2020; ^29^Brüggemann et al., 1998; ^30^Ward and Schroeder, 1994; ^31^Pottosin et al., 2003; ^32^Bihler et al., 2005; ^33^Chérel and Gaillard, 2019; ^34^Gobert et al., 2007; ^35^Ragel et al., 2019; ^36^Tang et al., 2020; ^37^Maathuis, 2011; ^38^Wang et al., 2013; ^39^Hamamoto et al., 2008; ^40^Dreyer et al., 2017; ^41^Chen et al., 2017; ^42^Feng et al., 2020; ^43^Rubio et al., 2020; ^44^Raddatz et al., 2020; ^45^Kwak et al., 2001; ^46^Wang Y. et al., 2018; ^47^Wang and Wu, 2013; ^48^Zhang et al., 2018; ^49^Wu L. B. et al., 2019; ^50^Shabala and Cuin, 2008; ^51^Ahmad et al., 2016; ^52^Liu et al., 2000; ^53^Zhao et al., 2007; ^54^Pottosin, 2015; ^55^Xie et al., 2005; ^56^Demidchik, 2014; ^57^Adem et al., 2020; ^58^Gaymard et al., 1998; ^59^Hosy et al., 2003; ^60^Corratgé-Faillie et al., 2017; ^61^Cuin et al., 2018; ^62^Buch-Pedersen et al., 2006, ^63^Su et al., 2019; ^64^Demidchik et al., 2010; ^65^Garcia-Mata et al., 2010; ^66^Zarza et al., 2019; ^67^Zarza et al., 2020; ^68^Demidchik and Maathuis, 2007; ^69^Demidchik and Tester, 2002; ^70^Essah et al., 2003; ^71^Keisham et al., 2018; ^72^Shabala et al., 2007a; ^73^Shabala et al., 2007b; ^74^Foreman et al., 2003; ^75^Zepeda-Jazo et al., 2011; ^76^Demidchik, 2018; ^77^Pottosin et al., 2018; ^78^Pei et al., 2000; ^79^Wu et al., 2010; ^80^Pottosin et al., 2014b; ^81^Pottosin et al., 2012; ^82^Velarde-Buendía et al., 2012; ^83^Yoda et al., 2006; ^84^Marina et al., 2008; ^85^Moschou et al., 2009; ^86^Rodríguez et al., 2009; ^87^An et al., 2012; ^88^Haruta and Sussman, 2012; ^89^Palmgren and Morsomme, 2018; ^90^Marty, 1999; ^91^Zhu et al., 2015; ^92^Grunwald et al., 2017; ^93^Hoffmann et al., 2020; ^94^Gjetting et al., 2020; ^95^Duby and Boutry, 2009; ^96^Bobik et al., 2010; ^97^Falhof et al., 2016; ^98^Yamauchi et al., 2016; ^99^Inoue and Kinoshita, 2017; ^100^Hoffmann et al., 2019; ^101^Yamauchi and Shimazaki, 2017; ^102^Zhao and Qin, 2004; ^103^Zepeda-Jazo et al., 2008; ^104^Janicka et al., 2018; ^105^Rubio et al., 2020; ^106^Bose et al., 2015b; ^107^Zhang et al., 2017; ^108^Xu et al., 2012; ^109^Yang et al., 2019; ^110^Xu et al., 2013; ^111^Kumari et al., 2019; ^112^Pottosin et al., 2014a; ^113^Pandolfi et al., 2010; ^114^Reggiani et al., 1992; ^115^Garufi et al., 2007; ^116^Janicka-Russak et al., 2010; ^117^Liu et al., 2006; ^118^Kabała and Kłobus, 2005; ^119^Bose et al., 2011; ^120^Bonza et al., 2016; ^121^Costa et al., 2017; ^122^Yang et al., 2017; ^123^Hilleary et al., 2020; ^124^Behera et al., 2018; ^125^Yu et al., 2018; ^126^García-Bossi et al., 2020; ^127^Bonza and De Michelis, 2011; ^128^Aslam et al., 2017; ^129^Schiøtt and Palmgren, 2005; ^130^Zhu et al., 2010; ^131^Sudha and Ravishankar, 2003.*

**FIGURE 1:**
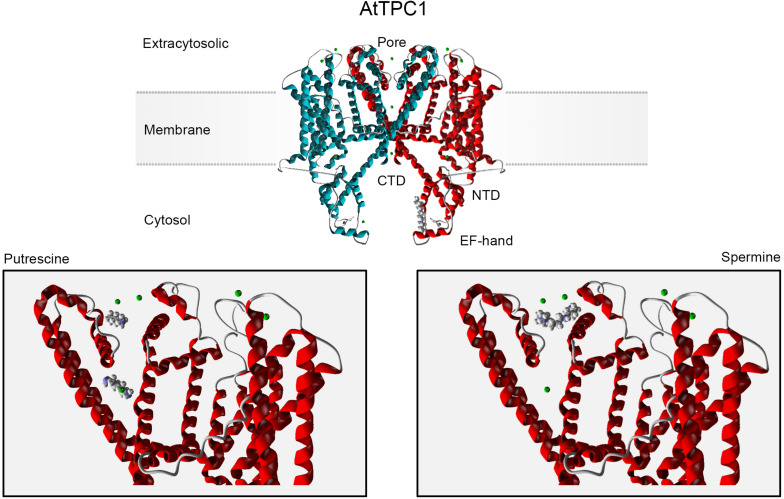
*In silico* docking analysis of the TPC1 interacting sites for polyamines. The AtTPC1 dimer is depicted in the upper panel. Monomers forming the channel are differently color-coded. Molegro Virtual Docker software (MVD; Molexus IVS: http://molexus.io/molegro-virtual-docker/) was employed using the AtTPC1 structure from the Protein Data Bank (PDB: https://www.rcsb.org/structure/5EJ1). The putrescine and spermine structures were obtained from PubChem (https://pubchem.ncbi.nlm.nih.gov/compound/1045; … 1103). The protein structure was optimized and cavities were detected for 5EJ1. Docking was performed for each cavity using the MolDock Optimizer as a search algorithm and the MolDock Score as a Scoring function. The lower panel corresponds to the docking prediction for PAs binding in the extracytosolic region. Two main binding sites for Put were predicted. The total binding energy for upper and lower binding conformations was −42.88 and −43.81, respectively (MolDockScore; MVD units). The Spm binding site partly overlaps the upper Put-binding site, with a higher binding energy (MolDockScore: −71.91 MVD units). Spm binding to this site involves two additional acidic residues, Asp 269 (−10.12 MVD units) and Glu 637 (−3.69 MVD units), which contribute by 19% into the total binding energy and determine the Spm orientation. An additional cytosolic binding site is predicted for Spm (upper panel, −73.43 MVD units), which overlaps with the EF2 hand regulatory Ca^2+^-binding site. Green spheres are permeable divalent cations, Ca^2+^ or Ba^2+^.

*In silico* analysis predicts two binding sites for Put within the TPC1 pore, one below and one above the filter (Figure 1, bottom left), in agreement with a multioccupancy block by this diamine (Pottosin, 2015). These sites overlap with the conducting route for permeable cations, implying their mutual competition. Longer Spm and Spd molecules partly share the external binding site with Put (Figure 1, bottom right), but their binding is much stronger. An additional binding site for Spm was predicted in the TPC1 cytosolic domain, overlapping with the regulatory Ca^2+^-binding site (Figure 1, top).

A suppression of the FV and SV channels by PAs is essential at any condition but is more important under salinity. FV and SV channels are unable to differentiate between K^+^ and Na^+^, thus mediating the Na^+^ leak from the vacuole (Pottosin and Dobrovinskaya, 2014). A futile vacuolar Na^+^ cycling has a rather high energy cost, imposing a penalty for plant performance (Shabala et al., 2020). The suppression of vacuolar FV and SV channels may also be relevant at conditions of K^+^ deprivation, preventing vacuolar reuptake of cytosolic K^+^ by FV and SV channels. Notably, the overexpression of the tonoplast K^+^ (Na^+^)/H^+^ antiporter NHX1, which mediates vacuolar K^+^ uptake, may be counterbalanced by a massive increase in Put under K^+^ deprivation (De Luca et al., 2018).

## Indirect Effects of Polyamines on the Plasma Membrane K^+^ Channels

In animal systems, PAs act as direct pore-plugging agents, underlying inward rectification of K^+^-selective Kir channels and exerting a voltage-dependent block of a variety of cation channels, including Na^+^ (lacking in plants) and ligand-gated channels (Williams, 1997; Guo and Lu, 2000; Huang and Moczydlowski, 2001). Cyclic nucleotides-gated and glutamate receptor channels in animal cells are blocked by PAs with a high affinity in a charge-specific manner. No data are available for their homologs, abundant in plants.

Plant voltage-dependent K^+^ channels belong to the Shaker family (Sharma et al., 2013). Their counterparts in animal cells are not blocked by the physiological concentrations of PAs. Inward rectifying K^+^ channels in guard cells are suppressed by endogenous PAs, which promote stomata closure under drought (Liu et al., 2000). An indirect inhibition was also reported for both inward- and outward-rectifying Shaker K^+^ channels in roots (Table 1). Membrane-bound PIP_2_ is a cofactor for numerous PM cation channels in animals (Suh and Hille, 2008) and inward- and outward-rectifying Shaker K^+^ channels in plants (Liu et al., 2005). PAs block animal inward rectifier Kir2.1 but also activate it by strengthening the interaction with PIP_2_ (Xie et al., 2005). PAs induced a rapid increase in PIP_2_ in *Arabidopsis* seedling, triggering a massive K^+^ efflux mediated by outward-rectifying GORK channels (Zarza et al., 2020). This mechanism appears to be specific for *Arabidopsis. In vivo* studies on pea and barley roots did not reveal any significant Spm-induced K^+^ efflux (Zepeda-Jazo et al., 2011; Velarde-Buendía et al., 2012).

## Pleiotropic Effects of Polyamines on H^+^ and Ca^2+^ Pumps

Operation of the PM-based H^+^- and Ca^2+^-ATPase pumps is central for responses to a variety of biotic and abiotic stresses (Table 1). An abrupt activation of H^+^ pumping occurs upon salt and hypertonic shock (Shabala and Cuin, 2008; Pandolfi et al., 2010; Bose et al., 2015a).

Upon long-term exposures, free and conjugated PAs could modify (either activate or inhibit) the activity of the PM H^+^- and Ca^2+^-ATPase pumps. This may be due to the changes in the protein expression, membrane composition/stability, and redox state (Srivastava and Rajbabu, 1983; Sudha and Ravishankar, 2003; Liu et al., 2005, 2014; Roy et al., 2005; Janicka-Russak et al., 2010; Du et al., 2015). Tonoplast H^+^ pumps, V-type ATPase and PPase, are only slightly affected by PAs, both upon direct application (Liu et al., 2004, 2006; Zhao and Qin, 2004) and after long-term exposure (Sun et al., 2002; Tang and Newton, 2005). Plant mitochondrial F-ATPase showed low sensitivity to PAs at physiological ionic conditions (Peter et al., 1981).

In pea and barley roots, PAs indiscriminately (Put∼Spm) induced eosin-sensitive Ca^2+^ efflux, pinpointing to the Ca^2+^-ATPase activation (Zepeda-Jazo et al., 2011; Velarde-Buendía et al., 2012; Pottosin et al., 2014b). Low millimolar concentrations of PAs stimulated the PM H^+^-ATPase activity in rice coleoptiles, but only Put can reach this concentration *in planta* (Reggiani et al., 1992). Spm and Spd caused a transient increase in a vanadate-sensitive H^+^ pumping in pea roots, whereas Put induced a sustained H^+^ efflux (Pottosin et al., 2014a). The general mechanism of the PM H^+^-ATPase activation involves a phosphorylation of a penultimate Thr, which promotes 14-3-3 protein binding and relief of the autoinhibition, while phosphorylation of other Ser and Thr residues in the C-terminal may either activate or inhibit the H^+^-ATPase (Falhof et al., 2016). Ca^2+^-dependent phosphorylation of Ser931 by SOS2-like kinase prevents the 14-3-3 protein binding, thus inhibiting the PM H^+^-ATPase (Fuglsang et al., 2007). PA-induced Ca^2+^-efflux may indirectly activate the H^+^-pumping *in vivo* by the local cytosolic Ca^2+^ depletion. Indeed, Put-induced stimulation of H^+^ pumping was suppressed by the Ca^2+^-pump inhibition, whereas Put did not affect the PM H^+^-pump *in vitro*. In addition, cytosolic Spm at higher concentration inhibited the H^+^ pumping by pea root PM vesicles (Pottosin et al., 2014a). Spm but not Spd or Put suppressed the H^+^ pumping in maize roots (Pandolfi et al., 2010). Contrary to this, Spm but no other PAs stimulated the PM H^+^-ATPase activity both in maize and *Arabidopsis* by a promotion of the 14-3-3 protein binding to the unphosphorylated C-terminal (Garufi et al., 2007). However, H^+^ efflux and H^+^-ATPase activity are not necessarily correlated. In its upregulated state, the PM H^+^-ATPase transports 1 H^+^ per 1 ATP, but this coupling may be loosened at high cytosolic cation concentration (Buch-Pedersen et al., 2006). Whether Spm may act as such an uncoupler remains to be elucidated. Also, Spm and Spd but not Put are able to compete with Mg^2+^ for binding to ATP, thus decreasing the ATPase activity, e.g., of a mitochondrial F-type ATPase (Igarashi et al., 1989). As Mg^2+^ and Spm binding sites in ATP are overlapped only partly, a ternary complex ATP-Mg^2+^-Spm can be formed, which is processed by an ATPase more rapidly than Mg-ATP (Meksuriyen et al., 1998).

## Effects of Polyamine Catabolites: ROS and GABA

A substantial part of PA effects may be attributed to their catabolites, and the balance of the PA synthesis and catabolization is stress-regulated and may define the net response (Moschou et al., 2012; Pottosin et al., 2014b; Pál et al., 2015; Gupta et al., 2016). The immediate product of PA oxidation by diamine- (DAO; for Put) and polyamine- (PAO; for Spm and Spd) oxidases is H_2_O_2_. It could be converted to a more aggressive hydroxyl radical (OH^•^) in the presence of the transition metals in the cell wall (Demidchik, 2014) and cytosol (Rodrigo-Moreno et al., 2013). These two ROS species target two types of conductances: (1) non-selective (Ca^2+^-permeable) also known as ROS-activated NSCC or ROSIC (Pei et al., 2000; Demidchik and Maathuis, 2007; Zepeda-Jazo et al., 2011) and (2) outward-rectifying K^+^-selective channels GORK and SKOR (Demidchik et al., 2010; Garcia-Mata et al., 2010). ROS generation upon PA catabolization in the apoplast is essential for the hypersensitive response to pathogen attack, wound-healing, stomata closure, and salinity-associated ROS signaling, among all (see Tavladoraki et al., 2012; Pottosin et al., 2014b). The source depends on the relative expression of amine oxidases: mainly DAO in dicots and mainly PAO in monocots (Moschou et al., 2008). Transmission of the external ROS signal to internal signaling is mediated by ROS-activated Ca^2+^ influx, positively fed back to ROS production due to a Ca^2+^ activation of the PM-bound NADPH-dehydrogenase (Takeda et al., 2008; Demidchik et al., 2018; Pottosin and Zepeda-Jazo, 2018). In pea roots, OH^•^ at lower and higher levels, respectively, induced a transient Ca^2+^ pumping by Ca^2+^-ATPase and a sustained Ca^2+^ influx via ROSIC (Zepeda-Jazo et al., 2011). OH^•^-induced Ca^2+^ pumping was potentiated by PAs, with Ca^2+^ efflux becoming sustained in pea roots in the presence of Spm (Zepeda-Jazo et al., 2011; Pottosin et al., 2012), whereas in barley, the OH^•^ and PA effects on Ca^2+^ pumping were roughly additive (Velarde-Buendía et al., 2012). PAs also potentiated the ROSIC-mediated K^+^ efflux (Zepeda-Jazo et al., 2011). This potentiation was much more pronounced in a salt-tolerant vs. salt-sensitive barley variety (Velarde-Buendía et al., 2012). In cereals such as barley and wheat, the magnitude of the ROS-induced K^+^ efflux correlates negatively with salt tolerance (Maksimović et al., 2013; Wang W. et al., 2018). However, ROS-induced K^+^ efflux by modified GORK channels can also result in a “metabolic switch” relocating more cell energy to stress defense (Demidchik, 2014; Shabala, 2017). A transient drop in the cytosolic K^+^ in a local region (e.g., root tip) *per se* could also serve as a stress signal (Shabala, 2017).

The γ-aminobutyric acid (GABA) in plants is produced via decarboxylation of glutamate or by a two-step Put catabolization. Increased DAO expression in response to abiotic stresses (Shelp et al., 2012) may lead to increased GABA production. Upon heavy metal and hypoxia, GABA production is fed back, promoting the PA biosynthesis and reducing their catabolization (Wang et al., 2014; Seifikalhor et al., 2020). GABA inhibits the malate efflux from roots by a direct binding to anion transporter ALMT1 (Ramesh et al., 2015). GORK contains a conserved GABA-binding motif, and GABA induces K^+^ efflux via GORK (Adem et al., 2020). GABA over-accumulating *Arabidopsis* mutant displayed increased activity of the PM H^+^ ATPase, a better control of the membrane potential and K^+^ retention/reduced ROS-induced K^+^ efflux from roots, and lower Na^+^ uptake, conferring salt tolerance (Su et al., 2019). GABA provokes a hyperpolarization, via either inhibition of anion efflux via ALMT or stimulation of the H^+^ pumping; it reduces ROS-induced K^+^ efflux but increases ROS-induced Ca^2+^ efflux from barley roots (Shabala et al., 2014; Gilliham and Tyerman, 2016). Thus, GABA may antagonize the effects of ROS overproduction under stresses, which increase K^+^ efflux and Ca^2+^ uptake.

Polyamines and their catabolism also rapidly upturn NO signaling, which targets multiple ion transporters. These include activation of the PM H^+^-ATPase, inhibition (by direct nitrosylation) of GORK channels, activation of Ca^2+^ influx across the PM, and Ca^2+^ release from intracellular stores. The latter could lead to Ca^2+^-dependent activation of the slow anion and inhibition of inward-rectifying K^+^ currents, provoking stomata closure (Pottosin and Shabala, 2014; Seifikalhor et al., 2019).

## Outlook

In contrast to Ca^2+^ and Mg^2+^, which act at a point, PAs with repeatedly space-distributed charges can form links between multiple binding anionic centers, which explains a higher binding affinity for longer polyamines in comparison to diamine Put, predicted by the docking analysis. Respective amino acids should be mutated to test whether they decrease the affinity of block by PAs and, if so, to verify whether this will be detrimental for plants’ performance under stress (e.g., salinity). In addition to pore block, long PA Spm likely interferes with cytosolic Ca^2+^ binding to the EF2 site (Figure 1). EF2 is critical for the AtTPC1 activation (Guo et al., 2016), whereas Ca^2+^ binding to EF1 likely has an allosteric effect, increasing Ca^2+^ affinity for EF2 (Demidchik et al., 2018). Spm binding along the EF2 loop would affect its mobility and Ca^2+^ affinity, thus potentially altering TPC1 gating.

There are several unresolved problems regarding PA effects on PM ion transporters. In plants, PAs affect two key PM ionotropic ATPases, Ca^2+^ and H^+^ pumps. Diverse and sometimes opposite effects on the H^+^-ATPase imply multiple and mostly indirect mechanisms. A rapid stimulation of Ca^2+^ pumping by PAs in barley and pea roots is worth to be explored in other plant species and *in vitro* studies with isolated Ca^2+^-ATPases. By analogy with their animal counterparts, glutamate receptors and cyclic nucleotide gated channels are plausible but unexplored targets for PAs in plants. In the latter case, in addition to direct effects, the signaling cascade involving PA catabolization–NO generation–activation of the adenylate cyclase-Ca^2+^ signal is worth to be explored (Jeandroz et al., 2013; Pottosin and Shabala, 2014). Overall, signaling by PAs needs to be considered in a close context with ROS, NO, and Ca^2+^ signaling.

Direct effects of free and conjugated PAs on individual ion transporters should be compared, and underexplored effects of cadaverine and thermospermine should be addressed. Also, our knowledge on PA traffic and subcellular compartmentalization remains fragmentary. Early studies suggested that the vacuolar PA concentration is lower than that in the cytosol (Pistocchi et al., 1987; DiTomaso et al., 1992). Taking into the account the dominant vacuolar volume, it implies that if one operates with an average tissue PA content, actual cytosolic and vacuolar PA concentrations are several times under- and overestimated, respectively.

Polyamines alleviate stress-induced suppression of photosynthesis in different ways, including a stabilization of the thylakoid ultrastructure, control of lipid composition, regulation of the expression, structure and oligomerization of photosynthetic membrane proteins, promotion of the chlorophyll biosynthesis, and improvement of the antioxidant activity (Hamdani et al., 2011; Hu et al., 2014; Li et al., 2015, 2018; Shu et al., 2015). However, to the best of our knowledge, the effects of PAs on ion transport across chloroplast membranes have not been addressed. Meanwhile, chloroplasts possess a variety of cation and K^+^-selective channels in the inner envelope and thylakoid membranes, which finely tune the photosynthesis (for a review, see Checchetto et al., 2013). No data are available on the effects of PAs on mitochondrial ion channels in plants. Plausible PA targets, based on published data on animal mitochondria, involve a mitochondrial Ca^2+^ uniporter (MCU), controlling mitochondrial Ca^2+^ homeostasis, metabolism, and cell death (Yan et al., 2015 and references therein; Wagner et al., 2016), a mitochondrial transition pore (Cui et al., 2020), and an ATP- and ROS-sensitive mito K_ATP_ channel, whose activity decreases ΔΨ and damps mitochondrial ROS production under salinity and drought (Trono et al., 2015).

## Data Availability Statement

The raw data supporting the conclusions of this article will be made available by the authors, without undue reservation.

## Author Contributions

IP, OD, and SS developed the concept. MO-A performed the docking analysis. IZ-J composed the table. IP and OD wrote the draft. All authors edited the final version.

## Conflict of Interest

The authors declare that the research was conducted in the absence of any commercial or financial relationships that could be construed as a potential conflict of interest.
